# The relationship between psychological conditions and recurrence of benign paroxysmal positional vertigo: a retrospective cohort study

**DOI:** 10.1186/s12883-023-03169-8

**Published:** 2023-03-31

**Authors:** Yuexin Shu, Nannan Liao, Fang Fang, Qiuling Shi, Ning Yan, Yaoyue Hu

**Affiliations:** 1 School of Public Health, Chongqing Medical University, Chongqing, 400016 People’s Republic of China; 2 University-Town Hospital of Chongqing Medical University, Chongqing, 401331 People’s Republic of China

**Keywords:** Benign paroxysmal positional vertigo, Recurrence, Anxiety, Insomnia, Obsessive–compulsive disorder

## Abstract

**Background:**

Psychological conditions have been found to be associated with an increased risk of incident benign paroxysmal positional vertigo (BPPV). However, much less is known on whether and how psychological conditions such as anxiety, insomnia and obsessive–compulsive disorder (OCD) affect the recurrence of BPPV.

**Methods:**

A retrospective cohort study of 2,612 outpatients and inpatients diagnosed with BPPV between September 2012 and August 2020. BPPV recurrence was followed up until February 2021. The Cox proportional hazard regression was used to analyze the association between psychological conditions and the risk of the first recurrence. Poisson regression was applied to analyze the association between psychological conditions and the number of recurrences in patients with at least one relapse.

**Results:**

During the follow-up, 391 patients had at least one BPPV recurrence. Female BPPV patients were more likely than male patients to experience relapses than male patients, but the characteristics of BPPV recurrence (number of recurrences and duration between recurrences) did not differ between men and women. After adjustment for sex, age and comorbidities, a heightened risk of first BPPV recurrence was found to be associated with anxiety (hazard ratio [HR]: 1.30, 95% confidence interval [CI]: 1.01, 1.68) and OCD (HR: 2.15, 95% CI: 1.31, 3.52). An increased risk of first BPPV recurrence associated with insomnia was only observed in male patients (HR: 2.22, 95% CI: 1.24, 3.98) but not in female patients (HR: 0.91, 95% CI: 0.63, 1.31). None of these psychological conditions were associated with the number of recurrences in patients who experienced recurrence.

**Conclusions:**

The presence of anxiety and OCD increased the risk of first BPPV recurrence, as well as insomnia for male patients. These psychological conditions were not associated with the number of BPPV recurrences. Diagnosis and treatment of these psychological conditions could be a useful strategy to prevent the recurrence of BPPV.

**Supplementary Information:**

The online version contains supplementary material available at 10.1186/s12883-023-03169-8.

## Introduction

Benign paroxysmal positional vertigo (BPPV) is an idiopathic vestibular disease caused by an inappropriate stimulation of the semicircular canal ampullae induced by free-floating otoconia from the utricle [1], which manifests with transient paroxysmal vertigo and nystagmus stimulated by changes in the head position [2]. As one of the most common vestibular disorders, BPPV was reported to have a lifetime prevalence of 2.4% [3], often occurring at age 50–60 with a female-to-male ratio of 2:1 to 3:1 [2]. After receiving canalith repositioning maneuver (CRM) – a preferred treatment of BPPV – more than 95% of cases can be resolved [4]. However, patients with BPPV often relapse after successful treatment, the recurrence rate of which is approximately 10%-30% [5, 6] and the 10-year recurrence could be as high as 50% [7]. Female patients are found to have a higher risk of recurrent BPPV (rBPPV) than their male counterparts [8–10]. Frequent recurrence of BPPV usually causes great inconvenience to patients’ daily living, thus substantially reducing patients’ quality of life and increasing the burden for both the patients and the healthcare system [11, 12].

Evidence has been piling up supporting the coexistence of vestibular diseases and psychological conditions [13–16]. A previous study showed that 37.5% of patients with vertigo were accompanied by anxiety and somatization disorder [17]. A case–control study reported that the prevalence of generalized anxiety disorder and obsessive–compulsive disorder (OCD) in BPPV patients was higher than that in the general population [18]. Another case–control study found that BPPV patients had higher levels of anxiety disorder, as well as an increased obsessive–compulsive attitude than their healthy matches [19]. Furthermore, a nationwide population-based cohort study showed that anxiety disorder was associated with a heightened incidence of BPPV [3]. Despite the lack of evidence on the pathophysiological relationship between psychological conditions and BPPV, a large number of studies have confirmed that patients with anxiety disorder have neuroinflammation and impaired neuroendocrine response, which are considered to be important pathophysiological factors of BPPV [20–23]. Psychological conditions may also influence the recovery from balance disorders, resulting in prolonged vertigo or dizziness [24, 25].

Most of the symptoms of BPPV occur during sleep at night or wake up in the morning [26]. A case–control study showed that the incidence of sleep disorders in BPPV patients was substantially higher than that in healthy controls [27]. A population-based cohort study demonstrated patients with chronic sleep disorders, compared to those with other types of non-apnea sleep disorders, had the highest risk of BPPV [28]. It is suggested that bad sleep leads to multiple head movements during the night, therefore dispose BPPV patients to a higher risk of relapse [29]. In addition, patients with sleep disorders are prone to ischemia, hypoxia and recurrent hypoxemia [30], and long-term chronic hypoxia can result in damage and aging of otolith organs and the falling off of otoliths, triggering the occurrence of BPPV in turn [31].

Few studies have examined the influence of psychological conditions on the recurrence of BPPV [3, 11, 32] and some of them reported no such association [33, 34]. A retrospective cohort study showed that compared to BPPV patients without anxiety, the effectiveness of the first treatment markedly reduced in those with anxiety and their risk of recurrence increased [11]. Two other cohort studies found that the prevalence of insomnia was higher in rBPPV patients (30.4%) than in their counterparts without a recurrence (13.2%) [29], and that poor sleep quality was an independent risk factor of BPPV recurrence [35]. To our best knowledge, no study investigated the association between OCD and BPPV recurrence.

Since previous studies have focused on the relationships between anxiety and BPPV recurrence but less on other psychological conditions, in this retrospective cohort study of BPPV patients, we investigated how various psychological conditions including anxiety, insomnia and OCD were associated with the risk of BPPV recurrence among patients who were diagnosed with BPPV for the first time.

## Materials and methods

### Study population

Medical records of 2,612 outpatients and inpatients diagnosed with BPPV in University-Town Hospital of Chongqing Medical University, Chongqing, China between September 2012 and February 2021 were extracted and reviewed. The cohort consisted of BPPV patients who were diagnosed for the first time between September 2012 and August 2020. The time of their first BPPV diagnosis was defined as the baseline. The follow-up for recurrence of BPPV started immediately after patients were diagnosed and ended in February 2021. The follow-up time ranged from 6.0 to 111.0 months (mean: 40.5, standard deviation: 24.6). In clinical practice, the neurologists followed the Bárány Society criteria to diagnose BPPV [36]. BPPV was diagnosed based on patient’s medical history and symptoms, as well as signs of positional nystagmus identified during the Dix–Hallpike test and/or the supine roll test. Differential diagnoses of vestibular migraine and vestibular paroxysmia were made based on the relevant diagnostic criteria for the characteristics of the episodic vertigos and symptoms [36, 37]. Transcranial Doppler ultrasonography was used to aid in identifying basilar migraine during vertigo attacks [38, 39]. An electroencephalogram was done to detect whether patients had the epileptiform waveform in the presence of vestibular paroxysms. In addition, Meniere's disease, vestibular paroxysmal labyrinthitis, otorrhea and other otorhinolaryngological diseases were excluded by consulting otolaryngologists. Patients with head trauma were not included in this study. Once patients were diagnosed with BPPV, they received the CRM treatment according to their involved semicircular canal: Epley’s maneuver was performed for the anterior semicircular canal BPPV and posterior semicircular canal BPPV, while the Barbecue maneuver was performed for the lateral semicircular canal BPPV. After the CRM treatment, all patients had a positional test to determine if the treatment was successful. Patients who showed no nystagmus on the positional test but still expressed residual dizziness were treated with the same maneuvers again until dizziness symptoms were fully resolved. In line with a previous study, we defined rBPPV as a recurrence after at least a 15-day symptom-free interval following the successful treatment [40]. Since patients might experience multiple recurrences of BPPV, we also calculated the total number of recurrences.

### Psychological conditions

When patients were diagnosed with BPPV for the first time by neurologists, their psychological conditions were assessed if they presented relevant symptoms. Patient’s level of anxiety was evaluated using Zung’s Self-rating Anxiety Scale (SAS) [41]. The SAS contains 20 items covering a variety of anxiety symptoms with responses on a 4-point scale which range from 1 (none, or a little of the time) to 4 (most, or all of the time). A raw score of 20–80 was derived first and then converted into an index score of 25–100 by dividing the raw score by 80 and then multiplying it by 100 [42]. Anxiety was determined by having an index score of 50 and over [13]. Insomnia was diagnosed in accordance with the Chinese Adult Insomnia Diagnosis and Treatment Guide (2017 Edition) [43]that adopts the International Classification of Sleep Disorders-3 diagnostic criteria [44]. Severity of OCD was assessed using the Yale Brown Obsessive–Compulsive Scale (Y-BOCS) [45, 46]. The Y-BOCS consists of 10 items pertaining to obsessions and compulsions, and each item rates from 0 (no symptoms) to 4 (extreme symptoms). OCD was determined if the patients had a total Y-BOCS score of 8 and over (sub-clinical: 0–7, mild: 8–15, moderate: 16–23, severe: 24–31, extreme: 32–40) [47]. BPPV patients who met the aforementioned diagnostic criteria had pertinent psychological conditions identified and recorded in their medical records.

### Comorbidity

Chronic diseases such as hypertension, cardiovascular disease, diabetes, and hypercholesterolemia may exacerbate the degeneration of the posterior labyrinth and facilitate the detachment of otoconia [33, 48, 49], leading to the occurrence and/or recurrence of BPPV. Therefore, it is important to control for chronic diseases when investigating the association between psychological conditions and recurrence of BPPV. Several comorbidities were identified from the medical records at the time when patients were first diagnosed with BPPV, including hypertension, atherosclerosis, diabetes, cerebral infarction, posterior circulation ischemia, cervical spondylosis, osteoporosis, and dyslipidemia.

### Statistical analyses

Student’s t-test was used to examine the differences in age between BPPV patients with recurrence and without, while chi-squared test and Fisher’s exact test were used for psychological conditions and comorbidity. Among rBPPV patients, differences in the number of recurrences, duration between diagnosis and first recurrence and durations between first and second recurrence were tested using chi-squared test for trend. The rate of rBPPV by psychological conditions was shown using Kaplan–Meier survival curve and tested using log-rank test. Cox proportional hazard regression was performed first to analyze the associations of anxiety, insomnia and OCD with the risk of the first BPPV recurrence. Then we analyzed the association between each psychological condition and the number of BPPV recurrences among rBPPV patients using Poisson regression. A* p* value less than 0.05 was considered statistical significance. Since we found an interaction between sex and insomnia on the risk of first BPPV recurrence (*p* value of Wald test: anxiety = 0.19, insomnia = 0.004, OCD = 0.24), we further stratified our sample by sex when analyzing the association between insomnia and first BPPV recurrence. No interaction was found between sex and psychological conditions on the number of BPPV recurrences (*p* value of Wald test: anxiety = 0.78, insomnia = 0.76, and OCD = 0.90). Data analyses were performed using R version 4.1.2 (R Core Team, 2022).

## Results

Table 1 describes the baseline characteristics of all BPPV patients, as well as those with and without recurrent BPPV. Among the total of 2,612 BPPV patients, 828 (31.7%) were men and 1784 (68.3%) were women. The mean age of the first BPPV diagnosis was 52.98 years. At baseline, 559 (21.4%) patients had anxiety, whereas 269 (10.3%) and 83 (3.2%) patients had insomnia and OCD, respectively. The most common comorbidity was cervical spondylosis (402 patients, 15.4%), followed by hypertension (248 patients, 9.5%), cerebral infarction (159 patients, 6.1%), and atherosclerosis (143 patients, 5.5%). Baseline characteristics of patients with anxiety, insomnia, and OCD separately are provided in Supplementary Table 1. Compared with patients without psychological conditions (*N* = 1,974), those with psychological conditions tended to be older, were more likely to be female (for those with anxiety and insomnia), and have cervical spondylosis; but they were less likely to have hypertension (Supplementary Table 1). It is of note that anxiety, insomnia, and OCD can co-occur. In our cohort, a total of 638 patients had at least one psychological condition at baseline. Among them, 306 (48.0%) and 79 (12.4%) had anxiety and insomnia only, respectively, while none had OCD only (Supplementary Table 2). Meanwhile, 170 (26.6%) patients had both anxiety and insomnia, 63 (9.9%) had both anxiety and OCD, and 20 (3.1%) had all three psychological conditions.

**Table 1:** Baseline characteristics of BPPV patients

	**Total BPPV patients (*N* = 2612)**	**BPPV recurrent during follow-up**		
		**No (*N* = 2221)**	**Yes (*N* = 391)**	***P* value**
**Age at diagnosis** (mean, IQR)	52.98 (38.38, 64.34)	52.42 (36.52, 63.96)	57.03 (46.16, 66.55)	< 0.001
**Sex**				0.030
Men	828 (31.7)	723 (32.6)	105 (26.9)	
Women	1784 (68.3)	1498 (67.4)	286 (73.1)	
**Anxiety (%)**				0.004
No	2053 (78.6)	1768 (79.6)	285 (72.9)	
Yes	559 (21.4)	453 (20.4)	106 (27.1)	
**Insomnia (%)**				0.137
No	2343 (89.7)	2001 (90.1)	342 (87.5)	
Yes	269 (10.3)	220 (9.9)	49 (12.5)	
**Obsessive–compulsive disorder (%)**				0.057
No	2529 (96.8)	2157 (97.1)	372 (95.1)	
Yes	83 (3.2)	64 (2.9)	19 (4.9)	
**Hypertension (%)**				0.761
No	2364 (90.5)	2008 (90.4)	356 (91.0)	
Yes	248 (9.5)	213 (9.6)	35 (9.0)	
**Atherosclerosis (%)**				0.792
No	2469 (94.5)	2101 (94.6)	368 (94.1)	
Yes	143 (5.5)	120 (5.4)	23 (5.9)	
**Diabetes (%)**				0.033
No	2521 (96.5)	2136 (96.2)	385 (98.5)	
Yes	91 (3.5)	85 (3.8)	6 (1.5)	
**Cerebral infarction (%)**				0.191
No	2453 (93.9)	2092 (94.2)	361 (92.3)	
Yes	159 (6.1)	129 (5.8)	30 (7.7)	
**Posterior circulation ischemia (%)**				0.861
No	2547 (97.5)	2166 (97.5)	381 (97.4)	
Yes	65 (2.5)	55 (2.5)	10 (2.6)	
**Cervical spondylosis (%)**				0.061
No	2210 (84.6)	1892 (85.2)	318 (81.3)	
Yes	402 (15.4)	329 (14.8)	73 (18.7)	
**Osteoporosis (%)**				0.674
No	2601 (99.6)	2212 (99.6)	389 (99.5)	
Yes	11 (0.4)	9 (0.4)	2 (0.5)	
**Dyslipidemia (%)**				0.700
No	2485 (95.1)	2111 (95.0)	374 (95.7)	
Yes	127 (4.9)	110 (5.0)	17 (4.3)	

p for Student’s t-test, chi-squared test and Fisher’s exact test

*IQR*: interquartile range

During follow-up, 391 (15.0%) had at least one recurrence of BPPV, while 2,221 (85.0%) did not experience any recurrence (Table 1). The age at first BPPV diagnosis of rBPPV patients was older than those with no recurrence (*p* < 0.001). Among the rBPPV patients, 286 were women (73.1%), the proportion of which was higher than those with no recurrence (67.4%, *p* = 0.030). Compared to BPPV patients with no recurrence (20.4%), a higher proportion of rBPPV patients had anxiety (27.1%, *p* = 0.004), but such difference was not found for insomnia or OCD. Among all comorbidities, a difference was only observed for diabetes between rBPPV patients (1.5%) and those without recurrence (3.8%, *p* = 0.033). In rBPPV patients, the number of BPPV recurrences, the duration between diagnosis of BPPV and the first recurrence and the duration between first and second recurrences did not differ by sex (Table 2). However, compared to rBPPV patients aged 60 and over, younger rBPPV patients tended to have fewer recurrences (*p* for trend = 0.004) but with a shorter interval between diagnosis and first recurrence (*p* for trend = 0.003).

**Table 2:** Characteristics of BPPV recurrences

	**BPPV patients with recurrence (*N* = 391, %)**					
	**Sex**			**Age**		
	Men (*N* = 105)	Women (*N* = 286)	P for trend	< 60 (*N* = 220)	≥ 60 (*N* = 171)	P for trend
**Number of recurrences**						
1	78 (74.3))	186 (65.0)	0.077	153 (69.5)	111 (64.9)	0.004
2	17 (16.2)	69 (24.1)		45 (20.5)	41 (24.0)	
3	6 (5.7)	19 (6.6)		15 (6.8)	10 (5.8)	
4 +	4 (3.8)	12 (4.2)		7 (3.2)	9 (5.3)	
**Duration between diagnosis and 1**^**st**^** recurrence**						
< 1 month	32 (30.5)	62 (21.7)	0.058	57 (25.9)	37 (21.6)	0.003
1–3 months	24 (22.9)	61 (21.3)		45 (20.5)	40 (23.4)	
4–6 months	7 (6.7)	38 (13.3)		28 (12.7)	17 (9.9)	
7–12 months	12 (11.4)	44 (15.4)		34 (15.5)	22 (12.9)	
> 12 months	30 (28.6)	81 (28.3)		56 (25.5)	55 (32.2)	
**Duration between 1**^**st**^** and 2**^**nd**^** recurrence**						
< 1 month	9 (33.3)	26 (26.0)	0.296	22 (32.8)	13 (21.7)	0.189
1–3 months	6 (22.2)	21 (21.0)		9 (13.4)	18 (30.0)	
4–6 months	2 (7.4)	13 (13.0)		6 (9.0)	9 (15.0)	
7–12 months	6 (22.2)	18 (18.0)		11 (16.4)	13 (21.7)	
> 12 months	4 (14.8)	22 (22.0)		19 (28.4)	7 (11.7)	

Chi-squared test for trend was used to compare differences between the two groups

**Fig. 1:**
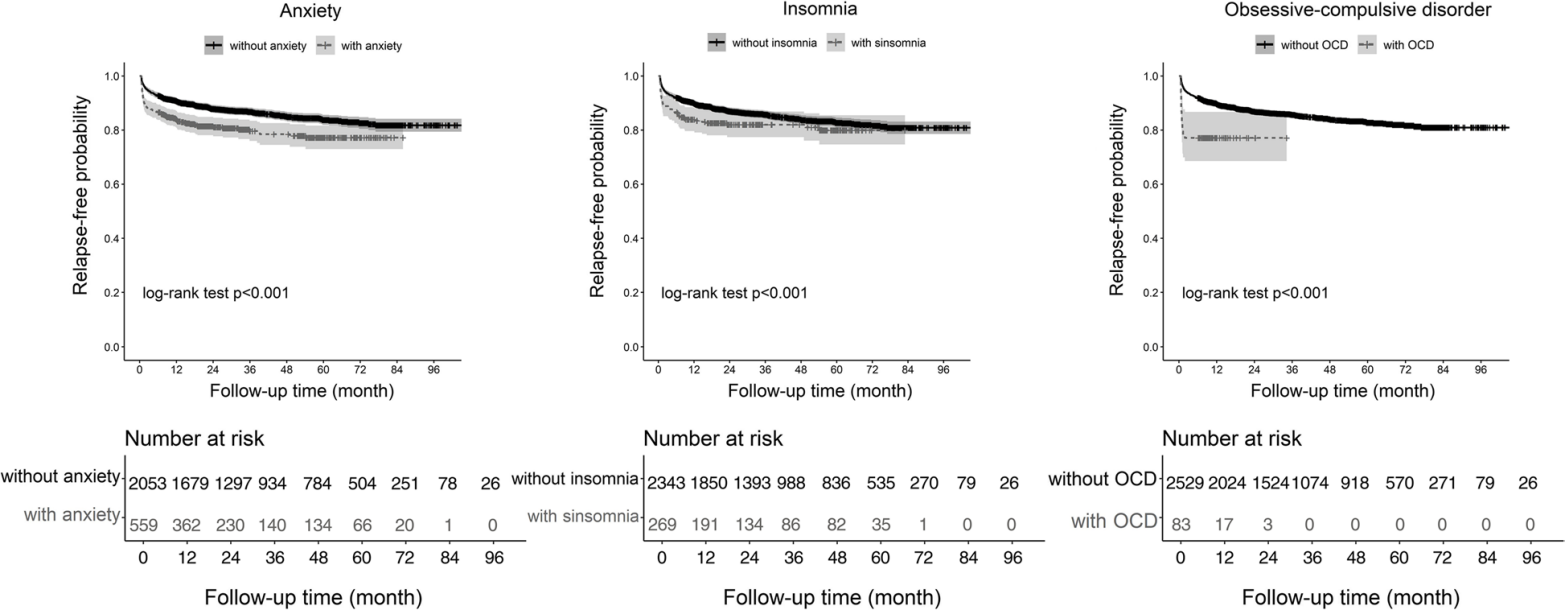
. Kaplan-Meier curves of the first BPPV recurrence for anxiety, insomnia, and OCD

The Kaplan–Meier curves of the first BPPV recurrence for anxiety, insomnia, and OCD are provided in Fig. 1. As shown in Fig. 1, the rate of the first recurrence differed by whether having anxiety (*p* for log-rank test < 0.001), insomnia (*p* < 0.001), and OCD (*p* < 0.001) or not. That is, BPPV patients with anxiety, insomnia, or OCD had a higher probability of BPPV relapse during follow-up than those without. It is of note that the first BPPV recurrence occurred in a short time interval since the first BPPV diagnosis among patients with OCD (Supplementary Table 3). Cox regression results showed that, after adjusting for sex and age (Model 1), the risk of first BPPV recurrence was higher for patients with anxiety (Table 3, hazard ratio [HR]: 1.43, 95% CI: 1.13, 1.79) and OCD (HR: 2.53, 95% CI: 1.59, 4.04) than those without such conditions. Further controlling for comorbidities at the time of BPPV diagnosis (Model 2), these associations were attenuated by 30% for anxiety (HR in Model 2: 1.30, 95% CI: 1.01, 1.68) and 25% for OCD (HR: 2.15, 95% CI: 1.31, 3.52). No association was found between insomnia and the risk of first BPPV recurrence (Model 1 in Table 3, HR: 1.20, 95% CI: 0.89, 1.62). Since anxiety, insomnia, and OCD could co-occur, we mutually adjusted for all psychological conditions and found that only OCD was associated with a heightened risk of first BPPV recurrence (HR in Model 2: 1.94, 95% CI: 1.16, 3.24). However, when comparing BPPV patients with anxiety or insomnia only to those without any psychological conditions, patients with anxiety only were found to have an elevated risk of first BPPV recurrence (Supplementary Table 4, HR in Model 2: 1.40, 95% CI: 1.03, 1.92), whereas no association was observed in patients with insomnia only (Supplementary Table 5).

**Table 3:** Cox regression results on the associations between psychological conditions and the first recurrence of BPPV

	**Model 1 (HR, 95% CI)**	**Model 2 (HR, 95% CI)**	**Model 1 (HR, 95% CI)**	**Model 2 (HR, 95% CI)**	**Model 1 (HR, 95% CI)**	**Model 2 (HR, 95% CI)**	**Model 1 (HR, 95% CI)**	**Model 2 (HR, 95% CI)**
**Anxiety**	1.43 (1.13, 1.79)^**^	1.30 (1.01, 1.68)^*^					1.30 (0.99, 1.70)	1.23 (0.92, 1.64)
**Insomnia**			1.20 (0.89, 1.62)	1.10 (0.81, 1.50)			1.00 (0.71, 1.39)	0.98 (0.70, 1.37)
**Obsessive–compulsive disorder**					2.53 (1.59, 4.04)^***^	2.15 (1.31, 3.52)^**^	2.06 (1.25, 3.41)^**^	1.94 (1.16, 3.24)^*^
**Sex**								
Men	1.00	1.00	1.00	1.00	1.00	1.00	1.00	
Women	1.26 (1.01, 1.58)^*^	1.27 (1.01, 1.59)^*^	1.28 (1.02, 1.61)^*^	1.28 (1.02, 1.60)^*^	1.32 (1.05, 1.65)^*^	1.31 (1.04, 1.64)^*^	1.28 (1.02, 1.60)^*^	1.28 (1.02, 1.61)^*^
**Age group**								
< 40	1.00	1.00	1.00	1.00	1.00	1.00	1.00	
40–49	1.29 (0.90, 1.85)	1.30 (0.91, 1.86)	1.31 (0.92, 1.88)	1.32 (0.92, 1.89)	1.30 (0.91, 1.86)	1.30 (0.91, 1.87)	1.29 (0.90, 1.85)	1.30 (0.91, 1.86)
50–59	1.68 (1.23, 2.29)^**^	1.69 (1.24, 2.30)^**^	1.73 (1.27, 2.36)^***^	1.72 (1.26, 2.34)^***^	1.73 (1.28, 2.36)^***^	1.72 (1.26, 2.35)^***^	1.67 (1.22, 2.28)^**^	1.68 (1.23, 2.30)^**^
60–69	1.69 (1.24, 2.30)^***^	1.77 (1.29, 2.43)^***^	1.78 (1.31, 2.42)^***^	1.83 (1.33, 2.51)^***^	1.77 (1.30, 2.40)^***^	1.83 (1.33, 2.51)^***^	1.71 (1.25, 2.33)^***^	1.79 (1.30, 2.46)^***^
≥ 70	2.13 (1.53, 2.97)^***^	2.25 (1.57, 3.22)^***^	2.24 (1.61, 3.12)^***^	2.34 (1.64, 3.33)^***^	2.25 (1.62, 3.12)^***^	2.34 (1.64, 3.34)^***^	2.15 (1.54, 2.99)^***^	2.24 (1.56, 3.21)^***^
**Hypertension**		0,84 (0.56, 1.26)		0.82 (0.55, 1.23)		0.83 (0.55, 1.24)		0.86 (0.57, 1.30)
**Cerebral infarction**		1.18 (0.79, 1.77)		1.17 (0.78, 1.74)		1.17 (0.78, 1.75)		1.19 (0.80, 1.79)
**Atherosclerosis**		1.08 (0.67, 1.75)		1.06 (0.65, 1.71)		1.08 (0.66, 1.75)		1.12 (0.69, 1.82)
**Posterior circulation ischemia**		0.95 (0.50, 1.79)		0.96 (0.51, 1.82)		0.93 (0,49, 1.76)		0.93 (0.49, 1.77)
**Cervical spondylosis**		1.23 (0.92, 1.64)		1.37 (1.05, 1.79)^*^		1.26 (0.95, 1.66)		1.16 (0.86, 1.56)
**Diabetes**		0.38 (0.17, 0.87)^*^		0.38 (0.17, 0.88)^*^		0.38 (0.17, 0.88)^*^		0.37 (0.16, 0.86)^*^
**Dyslipidemia**		0.87 (0.52, 1.50)		0.88 (0.53, 1.46)		0.89 (0.53, 1.47)		0.90 (0.54, 1.49)
**Osteoporosis**		0.96 (0.23, 4.02)		1.00 (0.24, 4.17)		1.00 (0.24, 4.20)		0.95 (0.23, 3.96)

*HR* hazard ratio, *CI* confidence interval

^*^ *p* < 0.05

^**^ *p* < 0.01

^***^ *p* < 0.001

Since we found an interaction between sex and insomnia on the risk of first BPPV recurrence (*p* value of Wald test: anxiety = 0.19, insomnia = 0.004, OCD = 0.24), we further stratified our sample by sex when analyzing the association between insomnia and first BPPV recurrence. After stratifying the sample by gender, male patients with insomnia had an increased risk of first BPPV recurrence (Model 1 in Table 4, HR: 2.55, 95% CI: 1.45, 4.50) than their counterparts without insomnia. Further adjustment of comorbidities attenuated this association by 21% (HR in Model 2: 2.22, 95% CI: 1.24, 3.98). Insomnia was not associated with the risk of first BPPV recurrence in women (HR in Model 1: 0.97, 95% CI: 0.68, 1.39). These results remained after mutually adjustment of all psychological conditions (HR in men: 2.01, 95% CI: 1.06, 3.81; women: 0.84, 95% CI: 0.57, 1.25).

**Table 4:** Cox regression results on the associations between insomnia and the first recurrence of BPPV

	**Model 1 (HR, 95% CI)**		**Model 2 (HR, 95% CI)**		**Model 1 (HR, 95%CI)**		**Model 2 (HR, 95% CI)**	
	**Men**	**Women**	**Men**	**Women**	**Men**	**Women**	**Men**	**Women**
**Insomnia**	2.55 (1.45, 4.50)^**^	0.97 (0.68, 1.39)	2.22 (1.24, 3.98)^**^	0.91 (0.63, 1.31)	2.03 (1.07, 3.83) *	0.85 (0.58, 1.26)	2.01 (1.06, 3.81) *	0.84 (0.57, 1.25)
**Anxiety**					1.52 (0.89, 2.59)	1.22 (0.89, 1.66)	1.25 (0.70, 2.23)	1.19 (0.85, 1.66)
**Obsessive–compulsive disorder**					0.97 (0.33, 2.83)	2.69 (1.52, 4.75) ***	0.85 (0.28, 2.57)	2.54 (1.42, 4.53) **
**Age group**								
< 40	1.00	1.00	1.00	1.00	1.00	1.00	1.00	1.00
40–49	1.45 (0.72, 2.95)	1.29 (0.85, 1.95)	1.45 (0.71, 2.94)	1.28 (0.84, 1.94)	1.45 (0.71, 2.94)	1.23 (0.81, 1.87)	1.45 (0.71, 2.95)	1.24 (0.81, 1.88)
50–59	1.69 (0.90, 3.17)	1.76 (1.24, 2.51)^**^	1.70 (0.90, 3.20)	1.73 (1.21, 2.48)^**^	1.62 (0.86, 3.05)	1.71 (1.20, 2.44) **	1.68 (0.89, 3.16)	1.71 (1.20, 2.45) **
60–69	1.83 (1.00, 3.36)	1.78 (1.24, 2.54)^**^	1.92 (1.03, 3.56)^*^	1.77 (1.22, 2.57)^**^	1.76 (0.96, 3.24)	1.68 (1.17, 2.42) **	1.89 (1.01, 3.51) *	1.72 (1.19, 2.50) **
≥ 70	2.38 (1.28, 4.43)^**^	2.24 (1.51, 3.31)^***^	2.75 (1.43, 5.30)^**^	2.18 (1.42, 3.35)^***^	2.30 (1.23, 4.30) **	2.17 (1.47, 3.22) ***	2.74 (1.42, 5.28) **	2.13 (1.38, 3.29) ***
**Hypertension**			0.61 (0.26, 1.45)	0.95 (0.60, 1.51)			0.62 (0.26, 1.48)	0.98 (0.62, 1.56)
**Cerebral infarction**			0.93 (0.41, 2.09)	1.30 (0.82, 2.06)			0.91 (0.40, 2.07)	1.32 (0.83, 2.10)
**Atherosclerosis**			1.02 (0.40, 2.64)	1.17 (0.67, 2.06)			1.03 (0.40, 2.67)	1.22 (0.69, 2.13)
**Posterior circulation ischemia**			1.30 (0.47, 3.58)	0.81 (0.35, 1.85)			1.26 (0.46, 3.50)	0.78 (0.34, 1.79)
**Cervical spondylosis**			1.37 (0.81, 2.32)	1.36 (1.00, 1.85)^*^			1.29 (0.72, 2.30)	1.14 (0.81, 1.60)
**Diabetes**			0.00 (0.00, 0.00)	0.49 (0.21, 1.13)			0.00 (0.00, 0.00)	0.48 (0.21, 1.12)
**Dyslipidemia**			0.86 (0.27, 2.76)	0.84 (0.47, 1.49)			0.88 (0.28, 2.83)	0.84 (0.47, 1.50)
**Osteoporosis**			7.20 (0.98, 53.06)	0.45 (0.06, 3.37)			6.20 (0.81, 47.42)	0.44 (0.06, 3.29)

*HR* hazard ratio, *CI* confidence interval

^*^ *p* < 0.05,

^**^ *p* < 0.01,

^***^ *p* < 0.001

No interaction was found between sex and psychological conditions on the number of BPPV recurrences (*p* value of Wald test: anxiety = 0.78, insomnia = 0.76, and OCD = 0.90). Among rBPPV patients, after controlling for age and sex (Model 1), the number of BPPV recurrences was not associated with anxiety, insomnia or OCD (Table 5). This observation did not change when comorbidities were further controlled, when all psychological conditions were mutually adjusted for, or when comparing patients with anxiety or insomnia only to patients with none of these psychological conditions (Supplementary Tables 6 and 7).

**Table 5:** Poisson regression results on the association between psychological conditions and number of BPPV recurrence

	**Model 1 (IRR, 95% CI)**	**Model 2 (IRR, 95% CI)**	**Model 1 (IRR, 95% CI)**	**Model 2 (IRR, 95% CI)**	**Model 1 (IRR, 95% CI)**	**Model 2 (IRR, 95% CI)**	**Model 1 (IRR, 95% CI)**	**Model 2 (IRR, 95% CI)**
**Anxiety**	1.18 (0.98, 1.41)	1.23 (0.99, 1.52)					1.17 (0.95, 1.44)	1.22 (0.97, 1.55)
**Insomnia**			1.03 (0.81, 1.32)	1.03 (0.80, 1.33)			0.94 (0.72, 1.22)	0.94 (0.72, 1.23)
**Obsessive–compulsive disorder**					1.25 (0.89, 1.75)	1.27 (0.88, 1.85)	1.13 (0.78, 1.63)	1.18 (0.81, 1.73)
**Sex**								
Men	1.00	1.00	1.00	1.00	1.00	1.00	1.00	1.00
Women	1.07 (0.88, 1.28)	1.06 (0.85, 1.23)	1.07 (0.89, 1.29)	1.07 (0.88, 1.29)	1.07 (0.89, 1.29)	1.06 (0.88, 1.28)	1.06 (0.88, 1.28)	1.06 (0.87, 1.27)
**Age group**								
< 40	1.00	1.00	1.00	1.00	1.00	1.00	1.00	1.00
40–49	1.06 (0.78, 1.44)	1.07 (0.79, 1.46)	1.08 (0.79, 1.47)	1.09 (0.80, 1.48)	1.08 (0.79, 1.47)	1.09 (0.80, 1.49)	1.07 (0.78, 1.45)	1.08 (0.79, 1.47)
50–59	1.16 (0.89, 1.51)	1.18 (0.90, 1.53)	1.20 (0.93, 1.56)	1.20 (0.92, 1.56)	1.19 (0.91, 1.54)	1.19 (0.91, 1.55)	1.16 (0.89, 1.51)	1.17 (0.90, 1.53)
60–69	1.15 (0.89, 1.50)	1.17 (0.89, 1.53)	1.18 (0.91, 1.54)	1.18 (0.90, 1.55)	1.18 (0.91, 1.53)	1.18 (0.90, 1.55)	1.16 (0.89, 1.51)	1.17 (0.89, 1.54)
≥ 70	1.15 (0.87, 1.52)	1.13 (0.84, 1.53)	1.20 (0.91, 1.59)	1.18 (0.88, 1.60)	1.20 (0.91, 1.58)	1.18 (0.88, 1.60)	1.15 (0.87, 1.53)	1.14 (0.84, 1.54)
**Hypertension**		0.92 (0.66, 1.28)		0.88 (0.64, 1.22)		0.89 (0.64, 1.23)		0.92 (0.66, 1.28)
**Cerebral infarction**		1.05 (0.76, 1.45)		1.05 (0.76, 1.45)		1.06 (0.76, 1.46)		1.05 (0.76, 1.46)
**Atherosclerosis**		1.00 (0.66, 1.52)		1.00 (0.65, 1.53)		1.01 (0.66, 1.55)		1.01 (0.66, 1.54)
**Posterior circulation ischemia**		1.06 (0.62, 1.81)		1.10 (0.64, 1.88)		1.07 (0.63, 1.84)		1.04 (0.61, 1.79)
**Cervical spondylosis**		0.90 (0.70, 1.15)		1.01 (0.81, 1.26)		0.96 (0.77, 1.21)		0.87 (0.68, 1.13)
**Diabetes**		1.29 (0.71, 2.34)		1.33 (0.73, 2.43)		1.33 (0.73, 2.41)		1.28 (0.70, 2.33)
**Dyslipidemia**		1.04 (0.66, 1.65)		1.09 (0.69, 1.72)		1.08 (0.68, 1.71)		1.04 (0.66, 1.65)
**Osteoporosis**		0.72 (0.17, 3.02)		0.62 (0.15, 2.61)		0.65 (0.16, 2.73)		0.75 (0.18, 3.15)

*IRR* incidence rate ratio, *CI* confidence interval

^*^ *p* < 0.05

^**^ *p *< 0.01

^***^ *p* < 0.001

## Discussion

In this retrospective cohort study of 2,612 BPPV patients, a higher proportion of female patients than male patients had recurrent BPPV during follow-up, but no sex difference was found in the risk of first recurrence or the number of recurrences in rBPPV patients. Anxiety and OCD were associated with a heightened risk of the first BPPV recurrence. An increased risk of first recurrence was also found in male patients with insomnia than their counterparts without, but no such association was observed in female patients. Among rBPPV patients, however, none of these psychological conditions was associated with the number of BPPV recurrences.

The recurrence rate of BPPV in our study was approximately 15%, which was in line with previous studies (10%-50%) [5–7]. A loss to follow-up nevertheless could occur that patients might have sought help in other hospitals when they experienced BPPV recurrences. However, the hospital that we obtained the data from is the main hospital for neurological diseases in the area, and the majority of the patients are residents nearby. As a result, the possibility that patients did not come back after they experienced a relapse is minimized. The majority of previous studies have demonstrated that most of the recurrent episodes of BPPV occur within the first year of diagnosis [6, 10, 50, 51]. However, Kansu and colleagues [52] showed that 53.8% of BPPV patients experienced the recurrence within the first two years, and two other studies found that the majority of recurrent episodes occurred within 4 years after the initial treatment [53, 54]. In line with Perez and colleagues [7] who reported that 50% of the BPPV recurrent episodes occurred within the first 6 months, 60.4% of the BPPV patients relapsed within six months after successful treatment in our study, and most of them only experienced one recurrence. Female BPPV patients have been reported to be at heightened risk of BPPV relapse than their male counterparts [6, 9, 10, 32], which may be related to menopause-related hormonal fluctuations and estrogen deficiency that might influence calcium metabolism, thus resulting in degeneration of otoconia [55]. This is consistent with our findings but contradicts the results of the study conducted by Kim and colleagues [50].

Two recent population-based retrospective cohort studies have demonstrated that patients with anxiety had a higher incidence risk of BPPV compared to their counterparts without anxiety [56, 57]. This could be related to the remarked reduction in the efficacy of the first CRM treatment in BPPV patients with anxiety resulting in an increased risk of recurrence within 6 months after the treatment [11]. The recovery of balance disorders including BPPV is a process of habituation and relearning that involves many different structures, mechanisms and activities such as neurophysiological adaptation, desensitization to dizziness sensations, recovery of automaticity in the perception and control of orientation, all of which are affected by psychological factors [24]. Anxiety thus could influence the recovery process of BPPV and leads to long-term vertigo or dizziness [24]. Another study also reported that the repeated existence of anxiety considerably increased the risk of failure in BPPV treatment [58]. In addition, the symptoms of BPPV are usually unpredictable and uncontrollable, which may cause uncertainty and fear of new symptoms [59]. Yardley and colleagues [60] summarized three distinctive clusters of worries in vertiginous patients: concern about losing control, fear of serious illness and anticipation of a severe attack of vertigo. Therefore, we speculate that psychological conditions may complicate and interfere with the process of habituation and coping, and increase the arousal of vertigo symptoms by amplifying autonomic symptoms [24]. We failed to find any interaction between anxiety and age on the risk of BPPV recurrence, suggesting that the effect of anxiety does not vary by age.

The mechanisms linking anxiety and BPPV remain unclear. Studies have shown that the detachment of otoliths in BPPV patients may be partly associated with oxidative stress and pro-inflammatory responses [61–63]. Anxiety is associated with a chronic low-grade inflammatory response and increased oxidative [21, 22, 64], which may further exacerbate the vestibular degeneration and lead to difficulty with the relocation and reabsorption of the dislodged otoliths [11]. The persistence of anxiety could elevate the likelihood of autonomic stimulation caused by vestibular function disorders [65]. Several epigenetic modulators (e.g., SIRT1, Cox2, FoxO, and P53) play important roles in the process of the detachment of otoliths and the recovery following a successful CRM treatment [20]. Persistent anxiety may also disrupt the balance of epigenetic modulation after a successful CRM treatment, resulting in a higher recurrence rate within a short period of time [11]. Neuroendocrine dysfunction caused by anxiety can also cause blood flow imbalance in the inner ear and influence the inner ear homeostasis, and thus affect the recovery of BPPV [56, 66].

Approximately 10% of BPPV patients exhibited insomnia in our study, which was lower than in another study with a proportion of 17% [29]. Wang and colleagues [35] found that poor sleep quality was associated with an increased risk of BPPV recurrence, although this finding was only drawn from 67 BPPV patients. Our study also observed an association between insomnia and first BPPV recurrence in male patients but not in female patients. Insomnia may trigger BPPV by inducing neuroendocrine dysfunction characterized by increased cortisol levels and sympathetic activity [28, 67, 68], as well as activation of inflammation of vestibular neurons [28, 69]. In our cohort, insomnia was similar in female rBPPV patients (35 out of 286, 12, 2%), male rBPPV patients (14 of 105, 13.3%) and female patients with no recurrence (178 out of 1320, 11.9%); but the proportion was much lower for male patients without recurrence (42 out of 723, 5.8%). The possible explanation may be that men are more vulnerable to the distress caused by the diagnosis and treatment of BPPV. Despite that the association between BPPV and OCD has not been adequately studied, Ketola and colleagues [70] recently showed that 46% of patients with vertigo had OCD. Although we found a heightened risk of first BPPV recurrence associated with OCD, the link remains unclear and needs further investigation.

Given the common co-occurrence of anxiety, insomnia, and OCD, when all psychological conditions were mutually adjusted for, the association between anxiety and the risk of first BPPV recurrence disappeared, while the association with OCD attenuated. However, when comparing BPPV patients with anxiety only to those with no psychological conditions, anxiety was found to be associated with an elevated risk of the first BPPV recurrence. This was likely due to the relatively strong correlation between the three psychological conditions, the mutual adjustment of which could lead to an over-adjustment.

In our study, none of the psychological conditions was found to be associated with the number of BPPV recurrences among rBPPV patients. The lack of association could be related to the treatments that patients received once being diagnosed with anxiety, insomnia and OCD which may take time to alleviate symptoms and recover. That is, these null findings may reflect the lagging effect of treating psychological conditions on BPPV recurrence.

Our study has several strengths and limitations. We used the inpatient and outpatient data for a long period from 2012 to 2021 in our examination of the association between psychological conditions and BPPV recurrence. We investigated not only anxiety but also insomnia and OCD, the latter of which were overlooked in the literature. Patients had a clear diagnosis of psychological conditions, particularly given the context that it is still considered a social stigma to be labelled having psychological conditions in China. However, given the nature of the data, detailed descriptive information on BPPV, such as residual dizziness and relapse after treatment, cannot be accessed. Although the hospital from which we obtained the data is the main hospital for neurological diseases in the area and the majority of the patients are local residents, it is possible that some of the patients visited other hospitals when they experienced recurrent BPPV. This lost to follow-up could lead to an underestimated BPPV recurrence rate as well as underestimated associations between psychological conditions and risk of BPPV recurrence. Nevertheless, it is possible that the health seeking-behavior may be different between those with mild symptoms and severe symptoms of BPPV, leading to an underestimation of the BPPV incidence. For BPPV patients with psychological conditions, the number of medical encounters for other symptoms such as dizziness may increase during the follow-up period, resulting in an over-estimation of BPPV recurrence among them and an over-estimation of the association between psychological conditions and BPPV recurrence. Furthermore, if the health-seeking behavior differs among those with psychological conditions than those without, our findings will be biased. The low prevalence of comorbid conditions in our study is likely because the diagnosis of those conditions might have been made by general practitioners rather than at the hospital. This may lead to an insufficient adjustment in the examination of the association between psychological conditions and BPPV recurrence. Since we extracted the medical records of BPPV patients from the hospital, we did not have information on some key factors such as body mass index and plasma Vitamin D3. Failure to control these factors in the analysis may lead to an over-estimation of the association between psychological conditions and BPPV recurrence.

To sum up, the presence of anxiety and OCD increased the risk of the first BPPV recurrence, while a heightened risk of the first recurrence associated with insomnia was only observed in male patients. None of these psychological conditions was associated with the number of BPPV recurrences. Diagnosis and treatment of psychologic conditions could be a useful strategy to prevent the recurrence of BPPV.

## Supplementary Information

**Additional file 1: Supplementary Table 1.** Baseline characteristics of BBPV patients with and without psychological conditions.** Supplementary Table 2.** Co-occurrence of psychological conditions.** Supplementary Table 3.** Survival table of the first BPPV recurrence in rBPPV patients with OCD (N=19).** Supplementary Table 4.** Cox regression results on the associations between anxiety and the first recurrence of BPPV, comparing BPPV patients with anxiety only with BPPV patients with no anxiety, insomnia or OCD.** Supplementary Table 5.** Cox regression results on the associations between insomnia and the first recurrence of BPPV, comparing BPPV patients with insomnia only with BPPV patients with no anxiety, insomnia or OCD.** Supplementary Table 6.** Poisson regression results on the associations between anxiety and the first recurrence of BPPV, comparing BPPV patients with anxiety only with BPPV patients with no anxiety, insomnia or OCD.** Supplementary Table 7.** Poisson regression results on the associations between insomnia and the first recurrence of BPPV, comparing BPPV patients with anxiety only with BPPV patients with no anxiety, insomnia or OCD.

## Data Availability

The raw data supporting the conclusions of this article will be made available by the University-Town Hospital of Chongqing Medical University, without undue reservation. The access of the data for research purpose should be submitted to and reviewed by the University-Town Hospital of Chongqing Medical University. (doc_yann@163.com). Not applicable.
