# Supplementary material for: The relationship between psychological conditions and recurrence of benign paroxysmal positional vertigo: a retrospective cohort study
Source: BMC Neurol. 2023 Mar 31;23:137. doi: 10.1186/s12883-023-03169-8 (PMC10064541; doi:10.1186/s12883-023-03169-8)
Supplement: Supplementary file 1 — Additional file 1: Supplementary Table 1. Baseline characteristics of BBPV patients with and without psychological conditions. Supplementary Table 2. Co-occurrence of psychological conditions. Supplementary Table 3. Survival table of the first BPPV recurrence in rBPPV patients with OCD (N=19). Supplementary Table 4. Cox regression results on the associations between anxiety and the first recurrence of BPPV, comparing BPPV patients with anxiety only with BPPV patients with no anxiety, insomnia or OCD. Supplementary Table 5. Cox regression results on the associations between insomnia and the first recurrence of BPPV, comparing BPPV patients with insomnia only with BPPV patients with no anxiety, insomnia or OCD. Supplementary Table 6. Poisson regression results on the associations between anxiety and the first recurrence of BPPV, comparing BPPV patients with anxiety only with BPPV patients with no anxiety, insomnia or OCD. Supplementary Table 7. Poisson regression results on the associations between insomnia and the first recurrence of BPPV, comparing BPPV patients with anxiety only with BPPV patients with no anxiety, insomnia or OCD. [file 12883_2023_3169_MOESM1_ESM.docx]

Supplementary Table 1. Baseline characteristics of BBPV patients with and without psychological conditions

|  | **BBPV patients with no psychological conditions (N=1974)** | **BPPV patients with psychological conditions** | | | | | |
| --- | --- | --- | --- | --- | --- | --- | --- |
|  |  | **Anxiety (N=559)** | **P value*** | **Insomnia (N=269)** | **P value*** | **OCD (N=83)** | **P value*** |
| **Age at diagnosis** (mean, IQR) | 51.23 (34.21, 63.26) | 57.96 (48.24, 66.46) | <0.001 | 57.10 (49.61, 66.05) | <0.001 | 57.18 (47.48, 65.54) | 0.002 |
| **Sex** |  |  | <0.001 |  | <0.001 |  | 0.921 |
| Men | 667 (33.8) | 144 (25.8) |  | 56 (20.8) |  | 29 (34.9) |  |
| Women | 1307 (66.2) | 415 (74.2) |  | 213 (79.2) |  | 54 (65.1) |  |
| **Hypertension (%)** |  |  | <0.001 |  | <0.001 |  | 0.010 |
| No | 1765 (89.4) | 527 (94.3) |  | 259 (96.3) |  | 82 (98.8) |  |
| Yes | 209 (10.6) | 32 (5.7) |  | 10 (3.7) |  | 1 (1.2) |  |
| **Atherosclerosis (%)** |  |  | 0.209 |  | 0.214 |  | 0.086 |
| No | 1860 (94.2) | 535 (95.7) |  | 259 (96.3) |  | 82 (98.8) |  |
| Yes | 114 (5.8) | 24 (4.3) |  | 10 (3.7) |  | 1 (1.2) |  |
| **Diabetes (%)** |  |  | 0.577 |  | 0.311 |  | 0.365 |
| No | 1902 (96.4) | 542 (97.0) |  | 263 (97.8) |  | 82 (98.8) |  |
| Yes | 72 (3.6) | 17 (3.0) |  | 6 (2.2) |  | 1 (1.2) |  |
| **Cerebral infarction (%)** |  |  | 0.439 |  | 0.622 |  | 0.239 |
| No | 1852 (93.8) | 530 (94.8) |  | 255 (94.8) |  | 81 (97.6) |  |
| Yes | 122 (6.2) | 29 (5.2) |  | 14 (5.2) |  | 2 (2.4) |  |
| **Posterior circulation ischemia (%)** |  |  | 0.015 |  | 1.000 |  | 0.033 |
| No | 1934 (98.0) | 537 (96.1) |  | 264 (98.1) |  | 78 (94.0) |  |
| Yes | 40 (2.0) | 22 (3.9) |  | 5 (1.9) |  | 5 (6.0) |  |
| **Cervical spondylosis (%)** |  |  | <0.001 |  | <0.001 |  | <0.001 |
| No | 1844 (93.4) | 298 (53.3)) |  | 181 (67.3) |  | 22 (26.5) |  |
| Yes | 130 (6.6) | 261 (46.7) |  | 88 (32.7) |  | 61 (73.5) |  |
| **Osteoporosis (%)** |  |  | 0.423 |  | 0.248 |  | 1.000 |
| No | 1968 (99.7) | 556 (99.5) |  | 267 (99.3) |  | 83 (100.0) |  |
| Yes | 6 (0.3) | 3 (0.5) |  | 2 (0.7) |  | 0 (0.0) |  |
| **Dyslipidemia (%)** |  |  | 0.366 |  | 0.050 |  | 0.033 |
| No | 1872 (94.8) | 536 (95.9) |  | 263 (97.8) |  | 83 (100.0) |  |
| Yes | 102 (5.2) | 23 (4.1) |  | 6 (2.2) |  | 0 (0.0) |  |

* Compared with BPPV patients with no psychological conditions; p for Student’s t-test, chi-squared test and Fisher’s exact test.

IQR: interquartile range

Supplementary Table 2. Co-occurrence of psychological conditions

|  | **BPPV patients with psychological conditions (N=638)** | |
| --- | --- | --- |
|  | **Only one psychological condition (N, %)** | **More than one psychological condition (N, %)** |
| Anxiety | 306 (48.0) |  |
| Insomnia | 79 (12.4) |  |
| OCD | 0 |  |
| Anxiety and insomnia |  | 170 (26.6) |
| Anxiety and OCD |  | 63 (9.9) |
| Anxiety, insomnia, and OCD |  | 20 (3.1) |

Supplementary Table 3. Survival table of the first BPPV recurrence in rBPPV patients with OCD (N=19)

| **Time in days** | **Number at risk** | **Number of Recurrence** | **Proportion surviving on this day** | **Cumulative relapse-free** |
| --- | --- | --- | --- | --- |
| 0 | 19 | 0 | 1.000 | 1.000 |
| 16 | 19 | 1 | 0.947 | 0.947 |
| 18 | 18 | 3 | 0.833 | 0.789 |
| 19 | 15 | 1 | 0.933 | 0.737 |
| 21 | 14 | 5 | 0.643 | 0.474 |
| 24 | 9 | 1 | 0.888 | 0.421 |
| 25 | 8 | 1 | 0.750 | 0.316 |
| 28 | 6 | 2 | 0.833 | 0.263 |
| 32 | 5 | 1 | 0.800 | 0.211 |
| 33 | 4 | 1 | 0.750 | 0.158 |
| 34 | 3 | 1 | 0.666 | 0.105 |
| 39 | 2 | 1 | 0.500 | 0.052 |
| 56 | 1 | 1 | 0.000 | 0.000 |

Supplementary Table 4. Cox regression results on the associations between anxiety and the first recurrence of BPPV, comparing BPPV patients with anxiety only with BPPV patients with no anxiety, insomnia or OCD

|  | **Model 1**  **(HR, 95% CI)** | **Model 2**  **(HR, 95% CI)** |
| --- | --- | --- |
| **Anxiety** | 1.43 (1.07, 1.90) | 1.40 (1.03, 1.92) |
| **Sex** |  |  |
| Men | 1.00 | 1.00 |
| Women | 1.33 (1.04, 1.70) | 1.34 (1.05, 1.71) |
| **Age group** |  |  |
| <40 | 1.00 | 1.00 |
| 40-49 | 1.35 (0.93, 1.96) | 1.36 (0.94, 1.98) |
| 50-59 | 1.62 (1.16, 2.24) | 1.65 (1.18, 2.30) |
| 60-69 | 1.64 (1.18, 2.28) | 1.73 (1.24, 2.43) |
| ≥70 | 2.16 (1.52, 3.07) | 2.33 (1.59, 3.42) |
| **Hypertension** |  | 0.89 (0.58, 1.35) |
| **Cerebral infarction** |  | 1.10 (0.71, 1.70) |
| **Atherosclerosis** |  | 1.23 (0.74, 2.03) |
| **Posterior circulation ischemia** |  | 0.75 (0.35, 1.60) |
| **Cervical spondylosis** |  | 1.06 (0.74, 1.51) |
| **Diabetes** |  | 0.40 (0.17, 0.91) |
| **Dyslipidemia** |  | 0.86 (0.51, 1.46) |
| **Osteoporosis** |  | 0.53 (0.07, 3.90) |

HR: hazard ratio; CI: confidence interval

Supplementary Table 5. Cox regression results on the associations between insomnia and the first recurrence of BPPV, comparing BPPV patients with insomnia only with BPPV patients with no anxiety, insomnia or OCD

|  | **Model 1**  **(HR, 95% CI)** | **Model 2**  **(HR, 95% CI)** |
| --- | --- | --- |
| **Insomnia** | 1.13 (0.65, 1.98) | 1.11 (0.63, 1.95) |
| **Sex** |  |  |
| Men | 1.00 | 1.00 |
| Women | 1.35 (1.04, 1.75) | 1.34 (1.03, 1.74) |
| **Age group** |  |  |
| <40 | 1.00 | 1.00 |
| 40-49 | 1.29 (0.87, 1.92) | 1.31 (0.88, 1.95) |
| 50-59 | 1.53 (1.09, 2.17) | 1.59 (1.12, 2.25) |
| 60-69 | 1.82 (1.29, 2.55) | 2.00 (1.41, 2.83) |
| ≥70 | 1.89 (1.28, 2.78) | 2.15 (1.41, 3.29) |
| **Hypertension** |  | 0.98 (0.62, 1.53) |
| **Cerebral infarction** |  | 1.09 (0.68, 1.75) |
| **Atherosclerosis** |  | 0.77 (0.41, 1.46) |
| **Posterior circulation ischemia** |  | 0.75 (0.30, 1.83) |
| **Cervical spondylosis** |  | 0.88 (0.53, 1.44) |
| **Diabetes** |  | 0.34 (0.12, 0.93) |
| **Dyslipidemia** |  | 0.81 (0.43, 1.51) |
| **Osteoporosis** |  | 1.86 (0.41, 8.37) |

HR: hazard ratio; CI: confidence interval

Supplementary Table 6. Poisson regression results on the associations between anxiety and the first recurrence of BPPV, comparing BPPV patients with anxiety only with BPPV patients with no anxiety, insomnia or OCD

|  | **Model 1**  **(IRR, 95% CI)** | **Model 2**  **(IRR, 95% CI)** |
| --- | --- | --- |
| **Anxiety** | 1.17 (0.97, 1.40) | 1.21 (0.97, 1.51) |
| **Sex** |  |  |
| Men | 1.00 | 1.00 |
| Women | 1.07 (0.89, 1.29) | 1.06 (0.88, 1.29) |
| **Age group** |  |  |
| <40 | 1.00 | 1.00 |
| 40-49 | 1.06 (0.78, 1.45) | 1.07 (0.78, 1.47) |
| 50-59 | 1.19 (0.90, 1.54) | 1.18 (0.91, 1.56) |
| 60-69 | 1.16 (0.89, 1.51) | 1.17 (0.89, 1.54) |
| ≥70 | 1.15 (0.87, 1.53) | 1.13 (0.83, 1.54) |
| **Hypertension** |  | 0.91 (0.66, 1.27) |
| **Cerebral infarction** |  | 1.05 (0.75, 1.47) |
| **Atherosclerosis** |  | 1.00 (0.65, 1.52) |
| **Posterior circulation ischemia** |  | 1.07 (0.62, 1.84) |
| **Cervical spondylosis** |  | 0.91 (0.70, 1.17) |
| **Diabetes** |  | 1.29 (0.71, 2.35) |
| **Dyslipidemia** |  | 1.04 (0.66, 1.65) |
| **Osteoporosis** |  | 0.76 (0.11, 5.51) |

IRR: incidence rate ratio; CI: confidence interval

Supplementary Table 7. Poisson regression results on the associations between insomnia and the first recurrence of BPPV, comparing BPPV patients with anxiety only with BPPV patients with no anxiety, insomnia or OCD

|  | **Model 1**  **(IRR, 95% CI)** | **Model 2**  **(IRR, 95% CI)** |
| --- | --- | --- |
| **Insomnia** | 1.06 (0.83, 1.36) | 1.12 (0.85, 1.48) |
| **Sex** |  |  |
| Men | 1.00 | 1.00 |
| Women | 1.05 (0.85, 1.29) | 1.04 (0.85, 1.29) |
| **Age group** |  |  |
| <40 | 1.00 | 1.00 |
| 40-49 | 1.06 (0.76, 1.49) | 1.08 (0.77, 1.51) |
| 50-59 | 1.21 (0.91, 1.62) | 1.22 (0.91, 1.63) |
| 60-69 | 1.19 (0.89, 1.58) | 1.21 (0.90, 1.63) |
| ≥70 | 1.25 (0.92, 1.71) | 1.25 (0.89, 1.75) |
| **Hypertension** |  | 0.88 (0.61, 1.27) |
| **Cerebral infarction** |  | 1.08 (0.76, 1.55) |
| **Atherosclerosis** |  | 0.91 (0.52, 1.56) |
| **Posterior circulation ischemia** |  | 1.30 (0.67, 2.53) |
| **Cervical spondylosis** |  | 0.82 (0.58, 1.17) |
| **Diabetes** |  | 1.16 (0.53, 2.52) |
| **Dyslipidemia** |  | 1.22 (0.69, 2.14) |
| **Osteoporosis** |  | 0.60 (0.14, 2.59) |

IRR: incidence rate ratio; CI: confidence interval
